# TGF-β1 inhibits human trophoblast cell invasion by upregulating kisspeptin expression through ERK1/2 but not SMAD signaling pathway

**DOI:** 10.1186/s12958-022-00902-9

**Published:** 2022-01-31

**Authors:** Lanlan Fang, Yang Yan, Yibo Gao, Ze Wu, Zhen Wang, Sizhu Yang, Jung-Chien Cheng, Ying-Pu Sun

**Affiliations:** Center for Reproductive Medicine, Henan Key Laboratory of Reproduction and Genetics, The First Affiliated Hospital of Zhengzhou University, 40, Daxue Road, Zhengzhou, Henan China

**Keywords:** TGF-β1, Kisspeptin, KISS1, Trophoblast cells, Invasion

## Abstract

**Background:**

Tightly regulation of extravillous cytotrophoblast (EVT) cell invasion is critical for the placentation and establishment of a successful pregnancy. Insufficient EVT cell invasion leads to the development of preeclampsia (PE) which is a leading cause of maternal and perinatal mortality and morbidity. Transforming growth factor-beta1 (TGF-β1) and kisspeptin are expressed in the human placenta and have been shown to inhibit EVT cell invasion. Kisspeptin is a downstream target of TGF-β1 in human breast cancer cells. However, whether kisspeptin is regulated by TGF-β1 and mediates TGF-β1-suppressed human EVT cell invasion remains unclear.

**Methods:**

The effect of TGF-β1 on kisspeptin expression and the underlying mechanisms were explored by a series of in vitro experiments in a human EVT cell line, HTR-8/SVneo, and primary cultures of human EVT cells. Serum levels of TGF-β1 and kisspeptin in patients with or without PE were measured by ELISA.

**Results:**

TGF-β1 upregulates kisspeptin expression in HTR-8/SVneo cells and primary cultures of human EVT cells. Using pharmacological inhibitor and siRNA, we demonstrate that the stimulatory effect of TGF-β1 on kisspeptin expression is mediated via the ALK5 receptor. Treatment with TGF-β1 activates SMAD2/3 canonical pathways as well as ERK1/2 and PI3K/AKT non-canonical pathways. However, only inhibition of ERK1/2 activation attenuates the stimulatory effect of TGF-β1 on kisspeptin expression. In addition, siRNA-mediated knockdown of kisspeptin attenuated TGF-β1-suppressed EVT cell invasion. Moreover, we report that serum levels of TGF-β1 and kisspeptin are significantly upregulated in patients with PE.

**Conclusions:**

By illustrating the potential physiological role of TGF-β1 in the regulation of kisspeptin expression, our results may serve to improve current strategies used to treat placental diseases.

## Background

The placenta is a unique organ in eutherian mammals which plays a pivotal role in reproduction. Placental development is marked by extravillous cytotrophoblast (EVT) cells invading the uterine wall and spiral arteries, replacing the cells of the vessel wall and creating a high-flow low-resistance vessel that ensures a continuous blood supply to the placenta throughout pregnancy [1]. Uncontrolled trophoblast cell invasion is associated with choriocarcinoma and hydatiform moles [2, 3]. In contrast, insufficient human trophoblast cell invasion into the uterine decidua and inadequate remodeling of the uterine vasculature leads to the development of preeclampsia (PE) which is a serious complication of pregnancy defined by high blood pressure and proteinuria [3, 4]. Therefore, trophoblast cell invasion must be tightly controlled, and a better understanding of the regulation of this process as well as the underlying molecular mechanisms will improve the diagnosis and treatment of pregnancy-related disorders.

Kisspeptin encoded by the KISS1 gene was first identified as a human melanoma metastasis suppressor gene [5]. Therefore, the kisspeptin is also known as metastin which belongs to the family of RF-amide peptides. Kisspeptin is a placenta-derived hormone in humans [6]. Kisspeptin exerts its biological function by binding to a G protein-coupled receptor, GPR54 [7]. Kisspeptin and GPR54 are highly expressed in the human placenta that plays important role in the regulation of placentation [8]. It has been shown that kisspeptin inhibits migration and invasion in primary human trophoblast cells and human EVT cell line, HTR-8/SVneo [9–12]. In the placenta of PE patients, the expression levels of kisspeptin are upregulated when compared to that in the placenta of uncomplicated pregnancies [13].

Transforming growth factor-beta1 (TGF-β1) belongs to the TGF-β superfamily which regulates diverse cellular functions and is involved in the regulation of various physiological and pathological processes [14]. TGF-β1 regulates cellular processes by binding to transmembrane type I and type II receptors [15]. In the canonical pathway, upon binding to receptors, TGF-β1 activates SMAD2/3 signaling pathways [16]. In non-canonical signaling pathways, TGF-β1 has been shown to act through MAPK and PI3K/AKT signaling pathways that are SMAD-independent [17]. TGF-β1 and its receptors are expressed in the human placenta [18]. A previous study has identified that the KISS1 gene is a mediator of TGF-β1-stimulated breast cancer cell invasion [19]. We and other groups have demonstrated that TGF-β1 inhibits the human EVT cell invasion [20–23]. Given the anti-invasive effect of kisspeptin in human EVT cells, whether kisspeptin expression is regulated by TGF-β1 and mediates the TGF-β1-inhibited cell invasion remains unknown. Therefore, in the present study, we examined the effect and the underlying molecular mechanisms of TGF-β1 on kisspeptin expression in human EVT cells. We also explored the role of kisspeptin in the TGF-β1-inhibited EVT cell invasion and compared the serum levels of TGF-β1 and kisspeptin between healthy and PE patients.

## Methods

### Antibodies and reagents

The KISS1 (#ab226786) antibody was purchased from abcam. The ALK5 antibody was purchased from Invitrogen (#PA5–78198). The phospho-SMAD2 (#3108), phospho-SMAD3 (#9520), SMAD2 (#3103), SMAD3 (#9523), and SMAD4 (#38454), phospho-ERK1/2 (#9106), ERK1/2 (#9102), phospho-AKT (#9271), and AKT (#9272) antibodies were purchased from Cell Signaling Technology. The α-tubulin (#sc-23948) antibody was purchased from Santa Cruz Biotechnology. The recombinant human TGF-β1 was obtained from R&D systems. The SB431542, LY294002, and EGF were obtained from Sigma. The U0126 was obtained from Cayman. The kisspeptin-10 was obtained from Tocris.

### Cell culture

The HTR-8/SVneo cell line was obtained from American Type Culture Collection through an official distributor in China (Beijing Zhongyuan Limited). HTR-8/SVneo is an SV40 large T antigen immortalized first-trimester short-lived extravillous trophoblast cell line [24]. Cells were cultured in a humidified atmosphere containing 5% CO_2_ and 95% air at 37 °C in Dulbecco’s modified Eagle’s medium/nutrient mixture F-12 Ham medium (DMEM/F-12; Gibco) supplemented with 10% charcoal/dextran-treated FBS (HyClone), 100 U/mL penicillin and 100 μg/mL streptomycin sulfate (Boster).

### Primary human EVT cell isolation and culture

The study received institutional approval and was carried out in accordance with the guidelines from the Zhengzhou University Research Ethics Board (#2020-KY-140). Human trophoblast cells were isolated from first trimester placental tissue explants as previously described [20, 23]. Briefly, chorionic villi were washed with cold medium and mechanically minced into 1–2 mm fragments. Fragments of the chorionic villi were allowed to adhere for 2–3 days, after which any non-adherent material was removed. These tissue explants were further cultured for 10–14 days to allow trophoblast outgrowth, during which the culture medium was changed every 2 days. Trophoblast cells were separated from the villous explants by brief trypsin digestion. Cells were cultured in a humidified atmosphere containing 5% CO_2_ and 95% air at 37 °C in Dulbecco’s modified Eagle’s medium/nutrient mixture F-12 Ham medium (DMEM/F-12) supplemented with 10% charcoal/dextran-treated FBS, 100 U/mL penicillin, and 100 μg/mL streptomycin sulfate. Individual primary cultures were composed of cells from one individual patient. Each experiment was repeated at least three times and each time used cells derived from different patients.

### Reverse transcription quantitative real-time PCR (RT-qPCR)

Total RNA was extracted with the TRIzol (Invitrogen) according to the manufacturer’s instructions. RNA (1 μg) was reverse-transcribed into first-strand cDNA with the iScript Reverse Transcription Kit (Bio-Rad Laboratories). Each 20 μL qPCR reaction contained 1X SYBR Green PCR Master Mix (Applied Biosystems), 60 ng of cDNA, and 250 nM of each specific primer. The following primers were used: KISS1, 5′-CAC TTT GGG GAG CCA TTA GA-3′ (sense) and 5′-CAG TAG CAG CTG GCT TCC TC-3′ (antisense); ALK5, 5′-GTT AAG GCC AAA TAT CCC AAA CA-3′ (sense) and 5′-ATA ATT TTA GCC ATT ACT CTC AAG G-3′ (antisense); SMAD4, 5′-TCC ACA GGA CAG AAG CCA TT-3′ (sense) and 5′-GTC ACT AAG GCA CCT GAC CC-3′ (antisense); Cx43, 5′-TAC CAA ACA GCA GCG GAGTT-3′ (sense) and 5′-TGG GCA CCA CTC TTT TGC TT-3′ (antisense); and GAPDH, 5′-GAG TCA ACG GAT TTG GTC GT-3′ (sense) and 5′-GAC AAG CTT CCC GTT CTC AG-3′ (antisense). qPCR was performed on an Applied Biosystems QuantStudio 12 K Flex system equipped with 96-well optical reaction plates. The specificity of each assay was validated by melting curve analysis and agarose gel electrophoresis of the PCR products. All of the RT-qPCR experiments were run in triplicate, and a mean value was used to determine the mRNA levels. Water and mRNA without RT were used as negative controls. Relative quantification of the mRNA levels was performed using the comparative Ct method with GAPDH as the reference gene and using the formula 2^–∆∆Ct^.

### Western blot analysis

Cells were lysed in cell lysis buffer (Cell Signaling Technology) supplemented with a protease inhibitor cocktail (Sigma). Equal amounts of protein were separated by SDS polyacrylamide gel electrophoresis and transferred onto PVDF membranes. After 1 h of blocking with 5% nonfat dry milk in Tris-buffered saline (TBS), the membranes were incubated overnight at 4 °C with primary antibodies diluted in 5% nonfat milk/TBS. Following primary antibody incubation, the membranes were incubated with appropriate HRP-conjugated secondary antibodies. Immunoreactive bands were detected using an enhanced chemiluminescent substrate (Bio-Rad Laboratories) and imaged with a ChemiDoc MP Imager (Bio-Rad Laboratories).

### Small interfering RNA (siRNA) transfection

To knock down endogenous ALK5, SMAD4, or KISS1 cells were transfected with 50 nM ON-TARGETplus SMARTpool siRNA targeting a specific gene (Dharmacon) using Lipofectamine RNAiMAX (Invitrogen). The siCONTROL NON-TARGETING pool siRNA (Dharmacon) was used as the transfection control.

### Invasion assay

Transwell cell culture inserts (8 μm pore size, 24 wells, BD Biosciences) were coated with 1 mg/mL growth factor-reduced Matrigel (BD Biosciences). Cells (1 × 10^5^ cells/insert) in DMEM/F12 medium supplemented with 0.1% FBS were incubated for 48 h against a gradient of 10% FBS. Noninvasive cells were removed with a cotton swab from the upper side of the membrane. Cells that penetrated the membrane were fixed with cold methanol. Cells were stained with crystal violet (0.5%, Sigma) for 30 min and subsequently washed thoroughly with tap water. Each experiment was performed with triplicate inserts. In each insert, five microscopic fields were photographed under an optical microscope, and the cell number was counted manually.

### ELISA assay

The present study received approval and was performed in accordance with the approved guidelines from the Zhengzhou University Research Ethics Board (#2020-KY-164). Written informed consent was obtained from all patients before collecting serum samples. TGF-β1 or kisspeptin protein levels in human serum samples were measured using an enzyme-linked immunosorbent assay (ELISA). The human TGF-β1 ELISA Kit (Elabscience, #E-EL-0162) and KISS1 ELISA Kit (Elabscience, #E-EL-H6099) were used following the manufacturer’s protocol. The analytical sensitivity of TGF-β1 ELISA was 0.1 ng/mL. Both intra-CV and inter-CV were < 10%. The analytical sensitivity of KISS1 ELISA was 18.75 pg/mL. Both intra-CV and inter-CV were < 10%.

### Statistical analysis

The results are presented as the mean ± SEM or mean ± SD of at least three independent experiments. All statistical analyses were analyzed by PRISM software. Multiple comparisons were analyzed using one-way ANOVA followed by Tukey’s multiple comparison test. For experiments involving only two groups, the results were analyzed by *t* test. A significant difference was defined as *p* < 0.05.

## Results

### The expression of kisspeptin is upregulated by TGF-β1 in human EVT cells

**Fig. 1:**
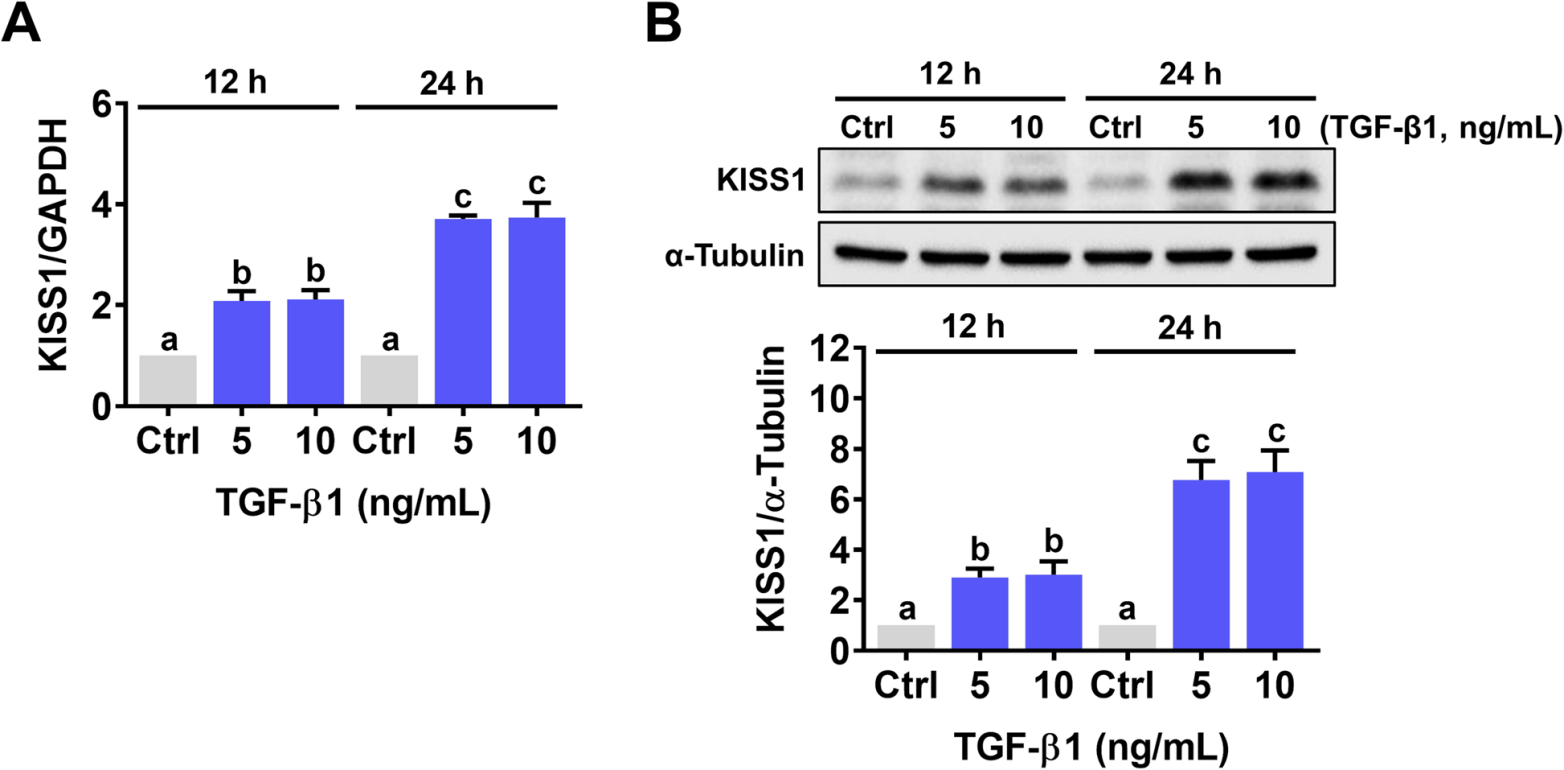
TGF-β1 upregulates kisspeptin expression in HTR-8/SVneo cells. **A** and **B** Cells were treated with 5 or 10 ng/mL TGF-β1 for 12 and 24 h. The kisspeptin mRNA levels (**A**) and protein levels (**B**) were examined by RT-qPCR and western blot, respectively. The results are expressed as the mean ± SEM of at least three independent experiments. Values that are statistically different from one another (*p* < 0.05) are indicated by different letters

**Fig. 2:**
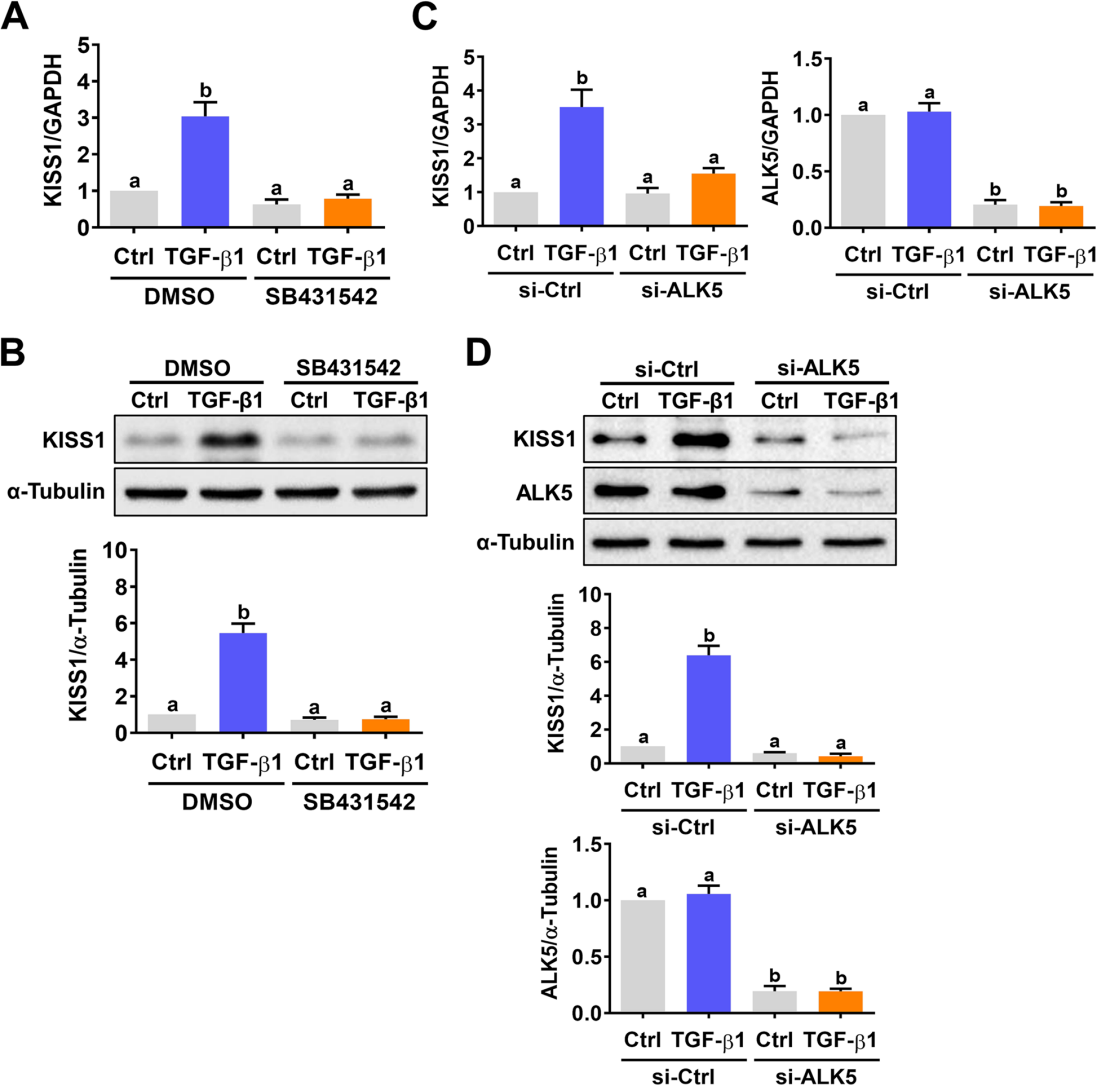
ALK5 mediates TGF-β1-induced kisspeptin expression in HTR-8/SVneo cells. **A** and **B** Cells were pretreated with vehicle control (DMSO) or 10 μM SB431542 for 1 h, and then treated with 5 ng/mL TGF-β1 for 24 h. The kisspeptin mRNA levels (**A**) and protein levels (**B**) were examined by RT-qPCR and western blot, respectively. **C** and **D** Cells were transfected with 50 nM control siRNA (si-Ctrl) or ALK5 siRNA (si-ALK5) for 48 h, and then treated with 5 ng/mL TGF-β1 for 24 h. The kisspeptin and ALK5 mRNA levels (**C**) and protein levels (**D**) were examined by RT-qPCR and western blot, respectively. The results are expressed as the mean ± SEM of at least three independent experiments. Values that are statistically different from one another (*p* < 0.05) are indicated by different letters

To examine the effect of TGF-β1 on kisspeptin expression in human EVT cells, HTR-8/SVneo human EVT cells were treated with 5 or 10 ng/mL TGF-β1 for 12 and 24 h. As shown in Fig. 1A, treatment with 5 or 10 ng/mL TGF-β1 for 12 h slightly increased the mRNA levels of kisspeptin. The significant induction of kisspeptin mRNA expression was observed after 24 h of TGF-β1 treatment. Treatments with 5 and 10 ng/mL TGF-β1 showed a comparable stimulatory effect on kisspeptin mRNA levels. Western blot further confirmed the stimulatory effect of TGF-β1 on kisspeptin protein levels in HTR-8/SVneo cells (Fig. 1B). SB431542 is a potent and specific TGF-β type I receptor/ALK5 inhibitor [25]. Pre-treatment with SB431542 abolished the TGF-β1-induced kisspeptin mRNA and protein levels in HTR-8/SVneo cells (Fig. 2A and B). To avoid unknown off-target effects of pharmacological inhibitor and further confirm the requirement of the ALK5 in the TGF-β1-induced kisspeptin expression, ALK5 specific siRNA was applied to knockdown ALK5 expression. As shown in Fig. 2C and D, transfection of HTR-8/SVneo cells with ALK5 siRNA significantly downregulated endogenous ALK5 mRNA and protein levels. Meanwhile, the stimulatory effects of TGF-β1 on kisspeptin mRNA and protein levels were abolished by the knockdown of ALK5.

### TGF-β1 induces kisspeptin expression in human EVT cells is SMAD-independent

**Fig. 3:**
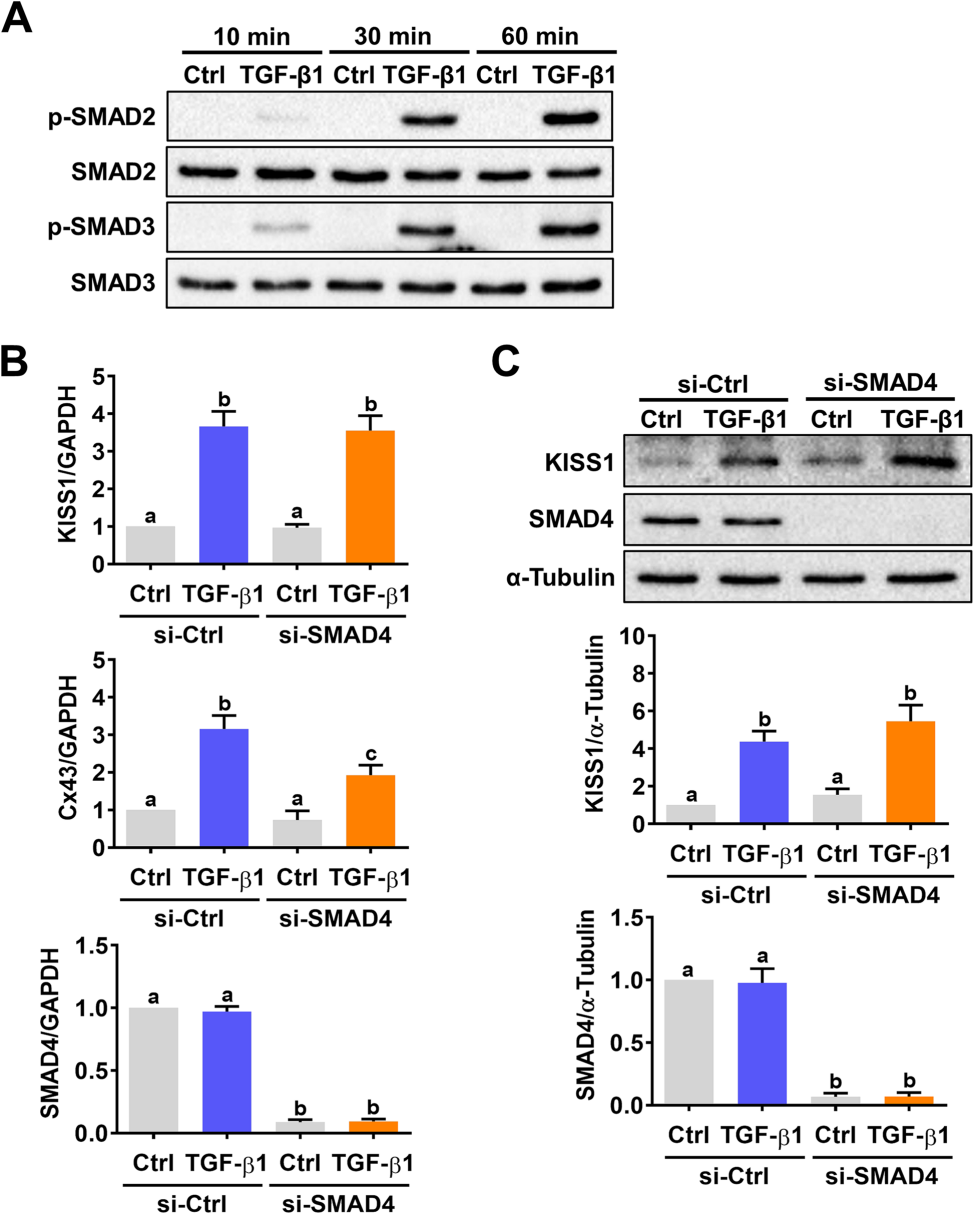
Activation of the SMAD signaling pathway is not involved in TGF-β1-induced kisspeptin expression in HTR-8/SVneo cells. **A** Cells were treated with 5 ng/mL TGF-β1 for 10, 30, and 60 min. The levels of phosphorylated and total forms of SMAD2 and SMAD3 were determined by western blot. **B **and** C** Cells were transfected with 50 nM control siRNA (si-Ctrl) or SMAD4 siRNA (si-SMAD4) for 48 h, and then treated with 5 ng/mL TGF-β1 for 24 h. The kisspeptin, Cx43, and SMAD4 mRNA levels (**B**) and protein levels of kisspeptin and SMAD4 (**C**) were examined by RT-qPCR and western blot, respectively. The results are expressed as the mean ± SEM of at least three independent experiments. Values that are statistically different from one another (*p* < 0.05) are indicated by different letters

In the canonical pathway, activation of ALK5 leads to phosphorylation of the downstream SMAD proteins, SMAD2/3. Phosphorylated SMAD2/3 then bind to the common-mediator SMAD4 and translocate into the nucleus where the SMAD complexes mediate TGF-β-regulated gene expression by binding to the SMAD-specific binding element in the promoter region [16]. As expected, treatment of HTR-8/SVneo cells with 5 ng/mL TGF-β1 for 10, 30, or 60 min induced phosphorylation of SMAD2 and SMAD3 indicating their activations (Fig. 3A). To examine whether SMAD signaling is required for the TGF-β1-induced kisspeptin expression, endogenous SMAD4 was knocked down by the siRNA transfection. Consistent with our previous study, knockdown of SMAD4 attenuated the TGF-β1-induced connexin 43 (Cx43) expression in HTR-8/SVneo cells [26] (Fig. 3B). As shown in Fig. 3B and C, transfection of HTR-8/SVneo cells with SMAD4 siRNA significantly downregulated endogenous SMAD4 mRNA and protein levels. Interestingly, the knockdown of SMAD4 did not affect the stimulatory effects of TGF-β1 on kisspeptin mRNA and protein levels. Collectively, these results indicate that the stimulatory effect of TGF-β1 on kisspeptin expression is mediated by the SMAD-independent pathways in human EVT cells.

### TGF-β1 induces kisspeptin expression in human EVT cells through the ERK1/2 signaling pathway

**Fig. 4:**
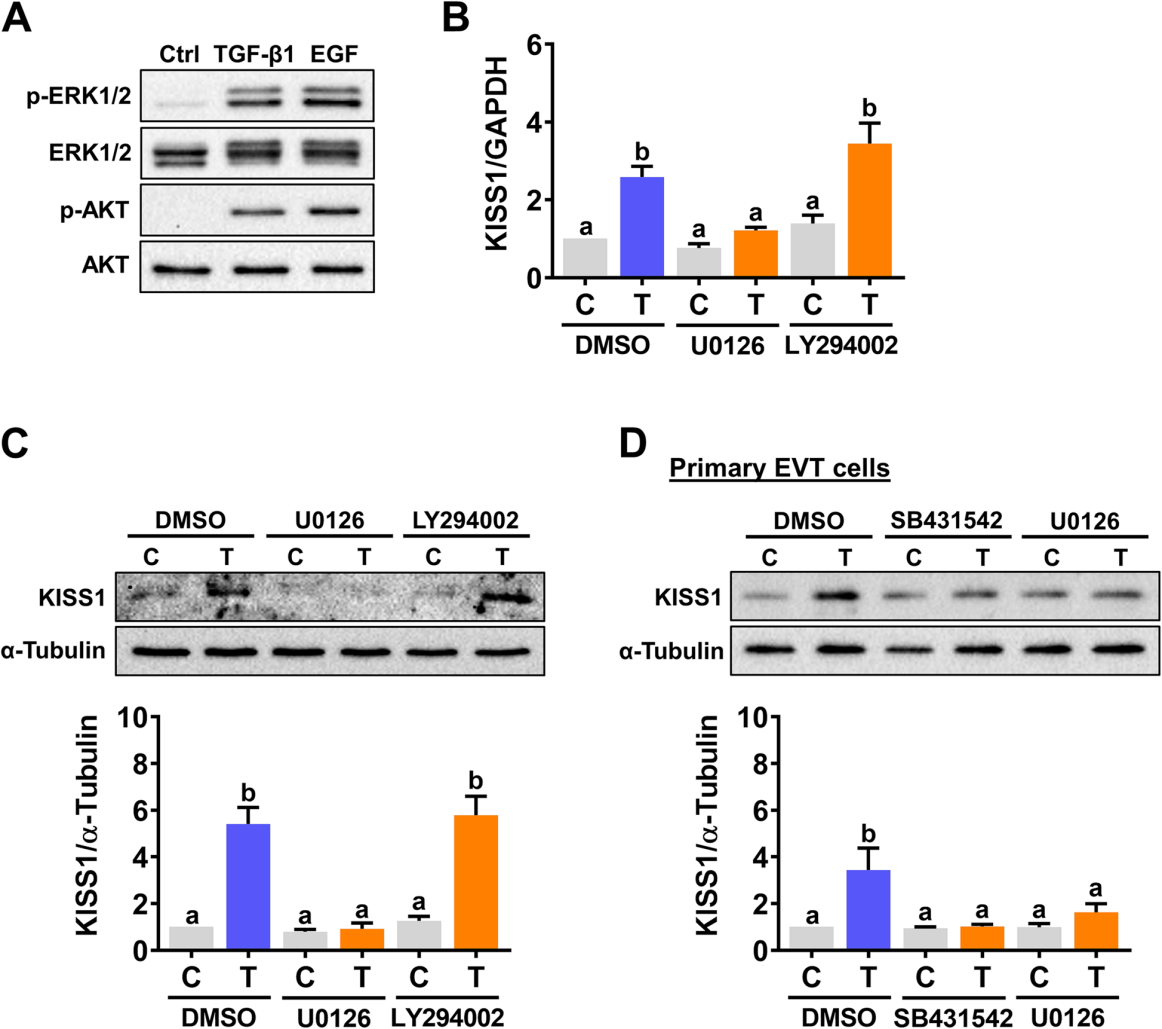
Activation of the ERK1/2 signaling pathway is required for TGF-β1-induced kisspeptin expression in human EVT cells. **A** HTR-8/SVneo cells were treated with 5 ng/mL TGF-β1 or 100 ng/mL EGF for 10 min. The levels of phosphorylated and total forms of ERK1/2 and AKT were determined by western blot. **B** and **C** HTR-8/SVneo cells were pretreated with vehicle control (DMSO), 5 μM U0126 or 5 μM LY294002 for 1 h, and then treated with 5 ng/mL TGF-β1 (T) for 24 h. The kisspeptin mRNA levels (**B**) and protein levels (**C**) were examined by RT-qPCR and western blot, respectively. **D** Primary cultures of human EVT cells were pretreated with vehicle control (DMSO), 10 μM SB431542 or 5 μM U0126 for 1 h, and then treated with 5 ng/mL TGF-β1 (T) for 24 h. The kisspeptin protein levels were examined by western blot. The results are expressed as the mean ± SEM of at least three independent experiments. Values that are statistically different from one another (*p* < 0.05) are indicated by different letters

ERK1/2 and PI3K/AKT are well-known non-canonical signaling pathways that mediate the biological function of TGF-β1. As shown in Fig. 4A, treatment of HTR-8/SVneo cells with 5 ng/mL TGF-β1 for 10 min activates both ERK1/2 and AKT signaling pathways. We used epidermal growth factor (EGF) as positive controls for the activations of ERK1/2 and AKT. To examine the involvement of the ERK1/2 and AKT signaling pathways in the TGF-β1-stimulated kisspeptin expression, the MEK inhibitor U0126, and PI3K inhibitor LY294002, were used to block their activations, respectively. As shown in Fig. 4B and C, blocking the activation of ERK1/2 attenuated the TGF-β1-induced kisspeptin mRNA and protein levels. Interestingly, inhibition of AKT did not affect the stimulatory effect of TGF-β1 on both kisspeptin mRNA and protein levels. Consistent with the results obtained from HTR-8/SVneo cells, treatment with 5 ng/mL TGF-β1 upregulated kisspeptin protein levels in primary human EVT cells. In addition, the stimulatory effect of TGF-β1 on kisspeptin protein levels in primary human EVT cells was abolished by the inhibition of ALK5 function and ERK1/2 signaling (Fig. 4D).

### Kisspeptin mediates TGF-β1-inhibited human EVT cell invasion

**Fig. 5:**
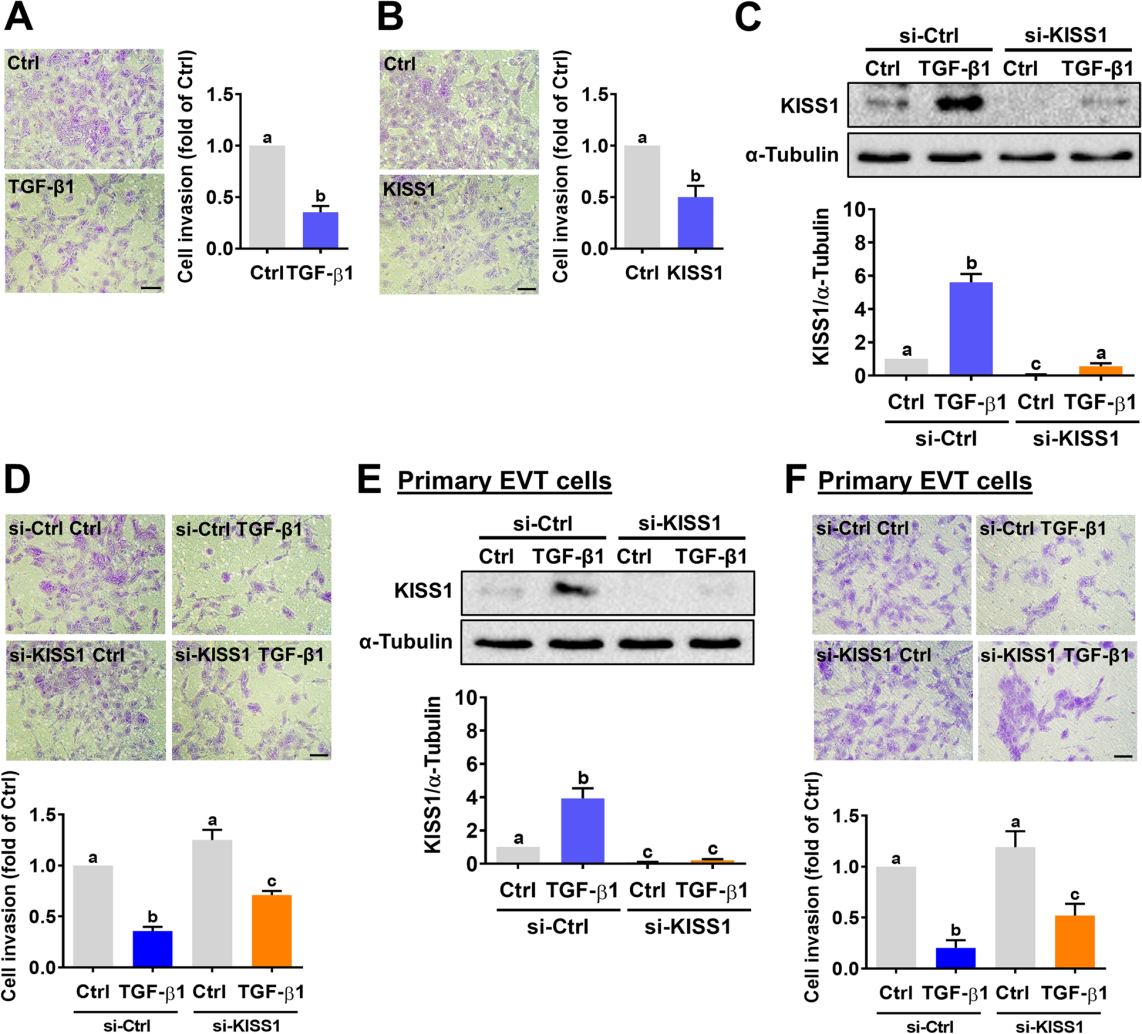
Kisspeptin mediates TGF-β1-suppressed human EVT cell invasion. **A** and **B** HTR-8/SVneo cells were treated with 5 ng/mL TGF-β1 (**A**) or 1 μM kisspeptin (**B**) and then seeded onto Matrigel-coated transwell inserts. The levels of cell invasiveness were examined by transwell invasion assay. **C** and **E** HTR-8/SVneo (**C**) and primary human EVT (**E**) cells were transfected with 50 nM control siRNA (si-Ctrl) or kisspeptin siRNA (si-KISS1) for 48 h, and then treated with 5 ng/mL TGF-β1 for 24 h. The kisspeptin protein levels were examined by western blot. **D** and **F** HTR-8/SVneo (**D**) cells and primary human EVT (**F**) cells were transfected with 50 nM control siRNA (si-Ctrl) or kisspeptin siRNA (si-KISS1) for 48 h and then treated with 5 ng/mL TGF-β1. The levels of cell invasiveness were examined by transwell invasion assay. Original magnification: 100x. The scale bar represents 50 μm. The results are expressed as the mean ± SEM of at least three independent experiments. Values that are statistically different from one another (*p* < 0.05) are indicated by different letters

Matrigel transwell invasion assay results showed that treatment with 5 ng/mL TGF-β1 or 1 μM kisspeptin significantly inhibited the invasiveness of HTR-8/SVneo cells (Fig. 5A and B). To examine the involvement of kisspeptin in TGF-β1-inhibited cell invasion, a siRNA-mediated knockdown approach was applied to block the expression of kisspeptin. As shown in Fig. 5C, transfection of HTR-8/SVneo cells with kisspeptin not only downregulated the basal endogenous kisspeptin protein levels but also abolished the TGF-β1-induced kisspeptin protein levels. Meanwhile, invasion assay results showed that the TGF-β1-inhibited HTR-8/SVneo cell invasiveness was attenuated by the knockdown of kisspeptin (Fig. 5D). Consistent with the results obtained from HTR-8/SVneo cells, knockdown of kisspeptin reduced the inhibitory effect of TGF-β1 on cell invasiveness in primary human EVT cells (Fig. 5E and F). Collectively, these results indicate that the TGF-β1-inhibited human EVT cell invasion is mediated by kisspeptin.

### Serum TGF-β1 and kisspeptin levels are upregulated in PE patients

**Fig. 6:**
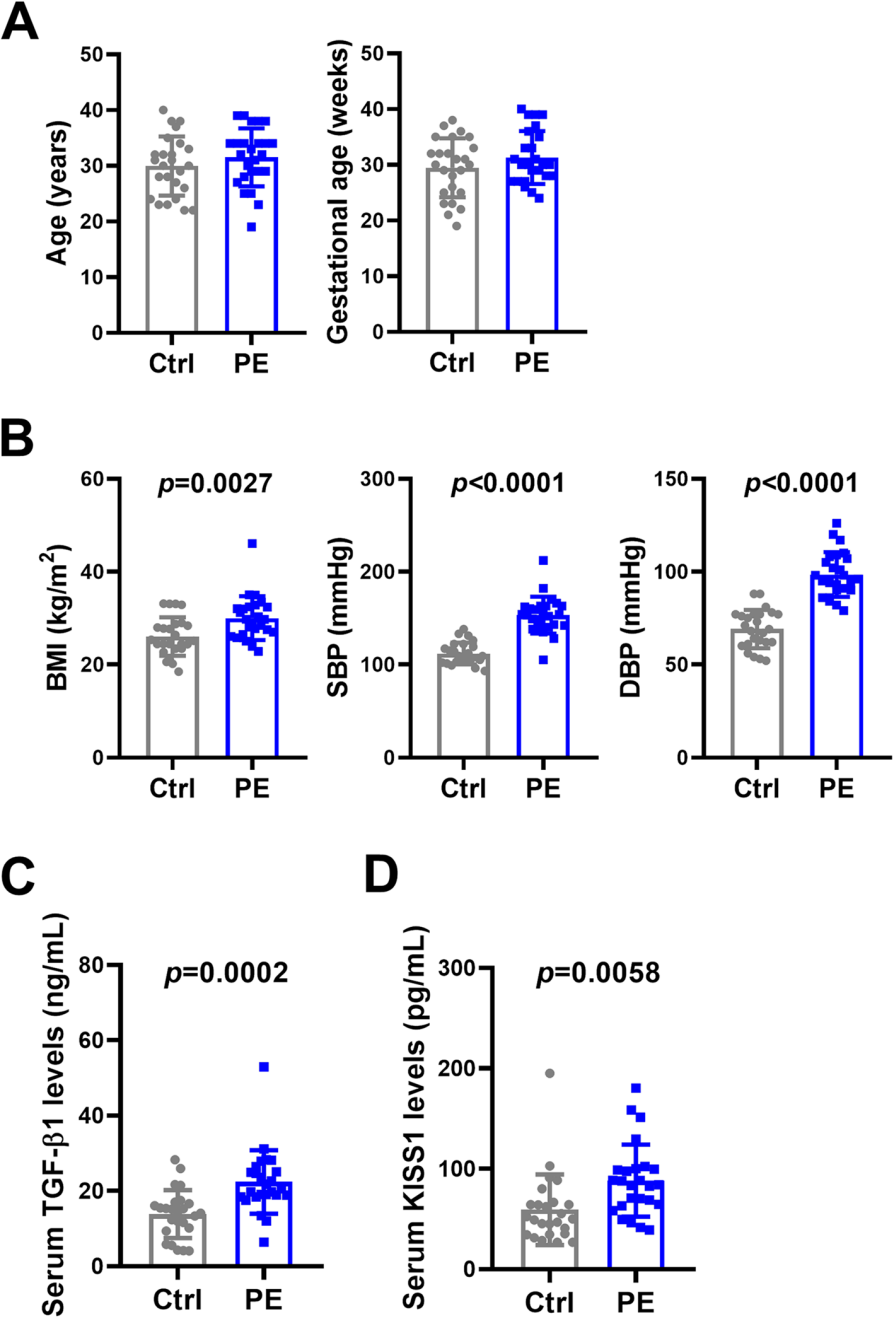
Serum levels of TGF-β1 and kisspeptin are upregulated in patients. **A** and **B** Serum samples were collected from 25 PE patients and 25 normal pregnant women of similar age and gestational age. The age and gestational age of the included patients are presented (**A**). BMI, systolic blood pressure (SBP), and diastolic blood pressure (DBP) were measured (**B**). **C** Serum levels of TGF-β1 were measured by ELISA. **D** Serum levels of KISS1 were measured by ELISA. The results are expressed as the mean ± SD

PE is a placental disease that can be caused by inadequate or incomplete trophoblast cell invasion which leads to insufficient blood flow to the uterus. Given the anti-invasive role of TGF-β1 and kisspeptin in human EVT cells, we examined the serum levels of TGF-β1 and kisspeptin in PE patients and compared them to those of women with normal pregnancies. Serum samples were collected from 25 PE patients and 25 normal pregnant women of similar age and gestational age (Fig. 6A). As expected, BMI, systolic blood pressure (SBP), and diastolic blood pressure (DBP) in PE patients were significantly higher than those in normal controls (Fig. 6B). ELISA results showed that both serum levels of TGF-β1 and kisspeptin were significantly upregulated in PE patients when compared to controls (Fig. 6C and D). Taken together, these results suggest that increased TGF-β1 expression in PE patients could lead to poor EVT cell invasion by upregulating kisspeptin expression.

## Discussion

The anti-invasive effects of TGF-β1 and kisspeptin have been reported in human EVT cells. However, whether kisspeptin is regulated by TGF-β1 and involved in TGF-β1-inhibited human EVT cell invasion remains unknown. In the present study, we reported that TGF-β1 upregulated kisspeptin expression in human EVT line, HTR-8/SVneo, and primary cultures of human EVT cells. Mechanically, we revealed that TGF-β1 induced kisspeptin expression through the SMAD-independent ERK1/2 signaling pathway. In addition, the TGF-β1-induced kisspeptin mediated the TGF-β1-inhibited human EVT cell invasion. Moreover, we showed that serum levels of TGF-β1 and kisspeptin were upregulated in PE patients.

It is known that kisspeptin is expressed in the hypothalamus where affects the hypothalamic-pituitary-gonadal axis and reproductive function by regulating gonadotropin-releasing hormone (GnRH) secretion [27]. In addition, kisspeptin plays important role in the pathogenesis of various human cancers [28, 29]. To date, most of the mechanisms that mediate the regulation of KISS1 gene expression are identified in the hypothalamic context of animal models and cancers. Although kisspeptin is highly expressed in the human placenta [30], the underlying molecular mechanism that mediates its expression there remains poorly defined. Previous studies have shown that estrogen, GnRH, neurokinin B, and glucocorticoids can stimulate KISS1 gene expression in human placental cells [31, 32]. In the present study, we showed that both mRNA and protein levels of kisspeptin in human EVT cells were upregulated by the treatment of TGF-β1. Interestingly, we revealed that SMAD signaling was not required for the TGF-β1-induced kisspeptin expression which is different from the previous findings that the stimulatory effect of TGF-β1 on kisspeptin is mediated by SMAD2 in triple-negative breast cancer cells [19]. These results suggest that the underlying mechanisms that mediate the TGF-β1-induced kisspeptin expression are in a cell-type dependent manner. In triple-negative breast cancer cells, melatonin induces kisspeptin expression by increasing the expression and transcriptional activation of GATA3 [33]. Activation of Ras-ERK1/2 cascade regulates GATA3-mediated gene expressions by increasing GATA3 protein stability [34]. Our results showed that activation of the ERK1/2 signaling pathway is required for the TGF-β1-induced kisspeptin expression. However, whether GATA3 is involved in TGF-β1-stimulated kisspeptin expression in human EVT cells remains unknown and warrants further investigations.

Matrix metalloproteinases (MMPs)-mediated extracellular matrix degradation or remodeling is a necessary event for the human EVT cell invasion [35, 36]. TGF-β1 has been shown to inhibit EVT cell invasion by downregulating the expression and activity of MMPs [21, 37]. Similarly, MMPs mediate kisspeptin-inhibited EVT cell invasion [10]. Here, we showed that knockdown of kisspeptin attenuated TGF-β1-inhibited HTR-8/SVneo cell invasion. Collectively, these results indicate that TGF-β1/kisspeptin/MMPs plays important role in regulating human EVT cell invasion. In rat ovaries, injection of human chorionic gonadotropin increases the kisspeptin mRNA levels, and this stimulatory effect is completely abolished by the inhibition of cyclooxygenase-2 (COX-2) [38]. These results indicate that kisspeptin is one of the downstream targets of COX-2/prostaglandins. Our previous study has shown that TGF-β1 inhibits HTR-8/SVneo cell invasion by upregulating the expression of COX-2 [39]. Therefore, although it needs to be confirmed, it seems plausible that COX-2 may mediate the TGF-β1-induced kisspeptin expression in human EVT cells. Interestingly, a TaqMan gene array shows that treatment of kisspeptin upregulates TGF-β1 mRNA levels in the first-trimester human trophoblast cells [10]. These findings together with our results suggest the reciprocal regulation of TGF-β1 and kisspeptin may exist in the human placenta.

Insufficient EVT cell invasion is one of the major characteristics of PE. Placental TGF-β1 levels are significantly higher in PE patients than controls [40]. In the present study, we showed that serum levels TGF-β1 are significantly elevated in patients with PE when compared to patients with normal pregnancy which is consistent with many previous studies [40–45]. However, few studies report that serum levels of TGF-β1 are not varied between control and PE patients [46–48]. One study reveals the opposite observation that serum levels of TGF-β1 are reduced in PE patients [49]. All these contradictory reports are probably due to differences in sample selection, collection, and preparation as well as differences in methodology. In addition to TGF-β1, our results showed that the serum levels of kisspeptin were increased in PE patients when compared to normal controls. It is interesting to note that the elevation of serum kisspeptin levels is conflicted with previous studies showing that serum kisspeptin levels are reduced in PE patients [50, 51]. Regardless of serum kisspeptin, the protein levels of kisspeptin are significantly upregulated in the placentas of PE patients [52]. Taken together, these clinical data support our in vitro experimental results that TGF-β1 inhibits human EVT cell invasion by upregulating kisspeptin expression.

## Conclusions

In summary, the present study demonstrates that exposure to TGF-β1 stimulates kisspeptin expression in HTR-8/SVneo and human primary EVT cells. This effect is mediated by the activation of the SMAD-independent ERK1/2 signaling pathway. In addition, the induction of kisspeptin is involved in TGF-β1-inhibited human EVT cell invasion. Moreover, both TGF-β1 and kisspeptin levels in serum are upregulated in PE patients. These results reveal the physiological role and underlying mechanism of TGF-β1 in the regulation of kisspeptin expression in human EVT cells and might help develop new strategies for the treatment of placental diseases.

## Data Availability

The data that support the findings of this study are available from the corresponding author upon reasonable request.

## Abbreviations

Cx43: Connexin 43
COX-2: Cyclooxygenase-2
DBP: Diastolic blood pressure
ELISA: Enzyme-linked immunosorbent assay
EGF: Epidermal growth factor
EVT: Extravillous cytotrophoblast
GnRH: Gonadotropin-releasing hormone
MMPs: Matrix metalloproteinases
PE: Preeclampsia
SBP: Systolic blood pressure
TGF-β1: Transforming growth factor-beta1
TBS: Tris-buffered saline
